# Adverse life experiences, mood symptoms, and quality of life in adults with down syndrome

**DOI:** 10.1007/s11136-026-04439-x

**Published:** 2026-11-12

**Authors:** Melissa R. Jenkins, Emily J. Hickey, Jessica L. Greenlee, Courtney E. Brothers, Dasoo M. Yoon, Ashlyn M. Barry, Emily K. Schworer, Benjamin L. Handen, Christy L. Hom, Amy E. Bodde, Lauren T. Ptomey, Sigan L. Hartley

**Affiliations:** 1 School of Social Work, University of North Carolina at Charlotte, Charlotte, NC 28223 USA; 2 Waisman Center, University of Wisconsin-Madison, Madison, WI 53705 USA; 3 School of Psychology, Lafayette College, Easton, PA 18042 USA; 4 Department of Human Development and Family Studies, University of Wisconsin-Madison, Madison, WI 53706 USA; 5 School of Education, University of Wisconsin-Madison, Madison, WI 53706 USA; 6 Division of Developmental and Behavioral Pediatrics, Cincinnati Children’s Hospital Medical Center, Cincinnati, OH 45229 USA; 7 Department of Pediatrics, University of Cincinnati College of Medicine, OH 45267 Cincinnati, USA; 8 Department of Psychiatry, University of Pittsburgh, Pittsburgh, PA 15213 USA; 9 Department of Psychiatry and Human Behavior, University of California, Irvine, Irvine, CA 92697 USA; 10 Department of Internal Medicine, University of Kansas Medical Center, Kansas City, KS 66103 USA

**Keywords:** Down syndrome, Disability, Adverse life experiences, Mental health, Quality of life

## Abstract

**Purpose:**

Life expectancy for people with Down syndrome (DS) has increased, creating a need to understand how to optimize later-life quality of life (QOL). In the general population, adverse life experiences (ALES) are thought to alter QOL via several mechanisms, including the presence of mood disorders. Little is known about whether ALES predict later-life QOL in adults with DS and, if so, whether this association is driven by increased mood symptoms.

**Methods:**

Data came from 133 adults with DS (*M*_age_ = 38.58 years, *SD* = 8.61) without dementia enrolled in the Alzheimer Biomarker Consortium-Down Syndrome. We used hierarchical linear regression to predict the effect of ALES and mood symptoms on QOL. We conducted an ordinary least squares path analysis to quantify the direct effect of ALES on QOL and the indirect effect through mood symptoms using 5000 bootstrap samples.

**Results:**

ALES was not a significant predictor of QOL. Mood symptoms were significantly negatively associated with QOL. The path analysis indicated that adults with DS who experienced more ALES also had more mood symptoms, and mood symptoms were associated with lower QOL. The confidence interval for the indirect effect was below zero, indicating that mood symptoms significantly affected the relationship between ALES and QOL. The direct effect of ALES on QOL was not significant.

**Conclusion:**

Mood symptoms are not simply a correlate of early adversity but an intervening variable through which the psychological and neurobiological effects of ALES may be expressed as poorer later-life QOL.

## Introduction

The life expectancy for people with Down syndrome (DS) has doubled since the 1980s and is now 60 years of age [1]. This increased longevity is attributed to policy and practice shifts in the education, services, and broader medical care of adults with DS, creating a need to understand how to optimize quality of life [2]. The World Health Organization defines quality of life as one’s “perception of their position in life in the context of the culture and value systems in which they live and in relation to their goals, expectations, standards and concerns” [3] (p.1405). For individuals with intellectual and developmental disabilities (IDD), Schalock and colleagues conceptualized quality of life as three higher order factors and eight underlying domains: (a) independence (i.e., personal development and self-determination); (b) social participation (i.e., interpersonal relations, social inclusion, and rights); and (c) wellbeing (i.e., emotional, physical, and material wellbeing) [4]. Quality of life research has primarily focused on the emotional wellbeing domain in adults with DS, while the rights domain is the least often assessed [5]. Schalock et al. [4] describe several potential mediators (e.g., employment status, attitudes) and moderators (e.g., gender, adaptive behavior) of quality of life in individuals with IDD. However, mood disorders were not specified despite being associated with lower quality of life among individuals with IDD [6]. Considering quality of life is a constellation of health and opportunity factors, the current study seeks to enhance understanding of the relationships among adverse life experiences (ALES), mood symptoms, and quality of life in adults with DS.

In the general population, adverse life experiences (ALES) are key determinants of quality of life [7–8]. The construct of ALES began with the Adverse Childhood Experiences Study [9], which defined ALES as exposure to childhood maltreatment and household challenges (e.g., parental incarceration) but has expanded to include social inequalities (e.g., poverty) and victimization outside the home and across the life course [10–11]. Approximately 63.9% of adults in the United States have experienced at least one ALE, with 17.3% experiencing at least four [12]. There is substantial evidence for the negative effect of ALES during childhood and adulthood on the quality of life of adults [9, 13–14]. One of the mechanisms posited to drive the link between adversity and a lower quality of life is the increased risk of mood disorders in later life [15–16].

There is evidence that individuals with IDD are at risk for a greater number of ALES relative to their neurotypical peers [17–21]. Individuals with IDD, including those with DS, are at a heightened risk of developing mood disorders following traumatic and stressful life events [22–23] ALES have been linked to DS regression disorder, defined as the sudden loss of functional skills, altered motor movements, and an increase in mood symptoms [24]. In a survey of caregivers, individuals with DS who had experienced DS regression disorder (*n* = 159) were more likely to report three or more ALES than those who had not experienced DS regression disorder (*n* = 178) [25]. To date, most research on adversity among individuals with DS examines the impact of these events proximally rather than early in or across the lifespan. For instance, recent distressing life events (e.g., loss of family or friends, hospitalizations, residential changes) have been associated with mood symptoms and functional decline in individuals with DS [26–31]. Further, poorer mental health has been linked to poorer quality of life and daily functioning in adults with DS [32–33]. However, little is known about whether ALES predicts quality of life in adults with DS and, if so, whether this association is at least partially explained through increased mood symptoms.

The goal of the current study was to examine the effect of ALES on quality of life in 133 adults with DS, and the indirect role of mood symptoms in this association. Based on prior work in the general population [15–16, 34–35], we hypothesized that (a) experiencing a higher number of ALES would be associated with lower quality of life, (b) experiencing a higher number of ALES would predict a higher level of mood symptoms, and (c) a higher level of mood symptoms would predict lower quality of life. We also investigated whether residential setting and level of independence were associated with the variables of interest given their importance to quality of life in individuals with IDD. Understanding the impact of adversity in adults with DS is important for supporting interventions that optimize quality of life.

## Methods

### Participants

Data came from 133 adults with DS (*M*_age_ = 38.58 years, *SD* = 8.61) enrolled in the Alzheimer Biomarker Consortium-Down Syndrome (ABC-DS) [36] who also agreed to be in an auxiliary lifestyle study. Participants were enrolled in four ABC-DS sites – University of Wisconsin-Madison, University of Pittsburgh, University of California-Irvine, and Washington University- St. Louis; ABC-DS was approved by Internal Review Boards. Eligibility criteria in ABC-DS included: (a) karyotype indicating full, mosaic, or translocation of chromosome 21, (b) 25 years of age or older, (c) mental age equivalent of at least 3 years of age, and (d) no health conditions that would interfere with brain imaging. We obtained consent from the adult with DS or their legal guardian (in which case, we obtained verbal assent from the adult with DS) in line with the Declaration of Helsinki. For the current analyses, we excluded adults with DS who had dementia.

### Study procedures

Participants and their study partner, who was a family member or caregiver who knew them well, attended a multi-day visit to complete ABC-DS and lifestyle study procedures with the study coordinator. The study partner reported on ALES, mood symptoms, and quality of life of the adult with DS as well as the larger battery of ABC-DS measures and procedures [30]. The study partner also reported health history, including medication history.

### Measures

#### Socio-demographics

Based on Schalock and colleagues’ contextualization of disability as a person-environmental interaction influencing quality of life outcomes, the control variables selected for analyses included four personal characteristics (chronological age in years, intellectual disability level, biological sex, and psychiatric medication use [i.e., anti-anxiety, anti-depressants, and/or mood stabilizers]) and two environmental factors (residential setting and level of independence). Intellectual disability level was based on medical records that documented IQ, or scores from the Stanford Binet Intelligence Test, Fifth Edition (SB5) [37] or the Kaufman Brief Intelligence Test, Second Edition (KBIT-2) [38]. Mental age equivalents were used to classify intellectual disability level as: mild = ≥ 9 years, moderate = 4–8 years, and severe = ≤ 3 years. Biological sex was coded: male = 1 and female = 0. Psychiatric medication was a composite variable coded: one or more medications = 1 and none = 0. Residential setting was coded: family caregiver = 1, group home = 2, independent living = 3. Level of independence was coded: no supervision = 0, minimum supervision = 1, 24-hour supervision = 2. Table 1 presents descriptive statistics for socio-demographics and mean scores for the CYW ACE-Q (number of ALES), ADAMS, and WHOQOL-DIS.

**Table 1:** Descriptive statistics of sociodemographics and study measures

Variable	Frequency (*N*) or *M*	Percent (%) or *SD*	Range
Sex			
Male	78	58.6	–
Female	55	41.4	–
Race/Ethnicity			
White, non-Hispanic	120	90.2	–
Non-White and/or Hispanic	13	9.8	–
Intellectual Disability			
Mild	58	43.6	–
Moderate	59	44.4	–
Severe	16	12.0	–
Karyotype			
Trisomy	123	93.9	–
Mosaicism	1	0.80	–
Translocation	6	4.6	–
Partial Trisomy	1	0.80	–
Psychiatric Medication			
No	97	72.9	–
Yes	36	27.1	–
Residence			
Family caregiver	93	69.9	–
Independent living	27	20.3	–
Group home	13	9.8	–
Level of Independence			
No supervision	12	9.0	–
Minimum supervision	92	69.2	–
24-hour supervision	29	21.8	–
Age (years)	38.58	8.61	25–61
ALES	2.27	2.23	0–10
ADAMS	8.46	8.27	0–40.44
QOL	71.05	13.55	32.14–96.43

*ALES* adverse life experiences, *ADAMS* anxiety, depression, and mood scale, *QOL* quality of life. *N* = 133

#### Adverse life experiences

The Center for Youth Wellness Adverse Childhood Experiences Questionnaire Teen (CYW ACE-Q) was used to measure ALES that occurred since the participant was born [39]. The CYW ACE-Q contains two sections: section one consists of 10 items from the original Adverse Childhood Experiences Study pertaining to abuse, neglect, and household dysfunction [8], and section two consists of nine items of additional early life stressors (e.g., parent or guardian death, foster care system involvement) identified by community stakeholders and experts. The CYW ACE-Q includes forms of adversity that may be relevant to study participants (e.g., serious medical procedure or life-threatening illness, treated badly because of disability or other identities). Study partners were instructed to report the total number of applicable questionnaire items per section, with ranges of 0 to 10 for section one and 0 to 9 for section two, which were aggregated into a total ALES score.

#### Mood symptoms

The Anxiety, Depression, and Mood Scale (ADAMS) [40] was used to measure mood symptoms that occurred within the past six months. The ADAMS consists of 28 items across five subscales: (a) manic/hyperactive behavior (e.g., has periods of over-activity, easily distracted), (b) depressed mood (e.g., sad, sleeps more than normal), (c) social avoidance (e.g., withdraws from other people, shy), (d) general anxiety (e.g., worried, experiences panic attacks), and (e) compulsive behavior (e.g., repeatedly checks items, engages in ritualistic behaviors). Response options for each scale item were: not a problem (0), mild problem (1), moderate problem (2), and severe problem (3). The total score ranged from 0 to 87, with higher scores indicating worse mood symptoms. The Cronbach’s alpha for the total score was 0.89 in the current sample, indicating strong internal consistency.

#### Quality of life

The World Health Organization Quality of Life-Disabilities Module (WHOQOL-DIS) was used to measure quality of life over a two-week period [41]. The 14-item measure consists of two general items (i.e., satisfaction with health, feel impacted by disability), three discrimination items (e.g., treated unfairly), three autonomy items (e.g., makes big decisions in life), and six inclusion items (e.g., satisfied with opportunities for social activities). Each item used a 5-point scale: not at all (1), a little (2), moderately (3), mostly (4), and totally (5). Items were summed, standardized, and transformed into a percent (see World Health Organization [42] for scoring details). The total score ranged from 0 to 100, with higher scores indicating better quality of life. The Cronbach’s alpha for the total score was 0.84 in the current sample, again indicating good internal consistency.

### Data analysis plan

Descriptive and inferential statistical analyses were conducted in SPSS v31.0.2.0 [43]. Bivariate correlations, one-way analysis of variance (ANOVA) models, and Welch’s tests (in cases of heteroscedasticity) were used to examine associations among the variables of interest (i.e., ALES, total ADAMS score, and total WHOQOL-DIS score) and socio-demographic variables. Eight participants had missing items on the WHOQOL-DIS (*n* = 4) and/or ADAMS (*n* = 6); however, more than > 80% of the items were completed, and thus item-mean imputation was used to fill in these missing values [44]. Of 158 participants, three (1.90%) had a clinical status of dementia and were excluded. An additional 22 (13.92%) did not have complete data on psychiatric medication use, residential setting, or level of independence. Participants with and without complete data did not significantly differ in their average number of ALES, *t*(153) = 0.459, *p* = .647; average total ADAMS score, *t*(153) = − 0.342, *p* = .733; and average total WHOQOL-DIS score, *t*(153) = 1.122, *p* = .264. They also did not differ with respect to covariates (age, sex, and intellectual disability level). Thus, we excluded 25 participants and included data from 133 participants in the analyses. ANOVA models also examined differences in ADAMS and WHOQOL-DIS scores by ALES groups: (a) no ALES, (b) one to three ALES, or (c) four or more ALES. These groupings are based on prior work indicating that four or more ALES is associated with high risk for adverse outcomes [9, 45].

We used a hierarchical linear regression model to predict the effect of ALES and mood symptoms on quality of life, adjusting for socio-demographic variables. We visually inspected partial-regression and normal probability plots to identify influential outliers and assess residual normality, respectively. We also used a Shapiro-Wilk test to statistically assess residual normality. We performed pathway analysis to quantify the direct effect of ALES on quality of life and the indirect effect through total ADAMS score. We conducted an ordinary least squares (OLS) path analysis and estimated a 95% bootstrap confidence interval for the indirect effect of total ADAMS score based on 5,000 bootstrap samples. To ensure no exposure-indirect effect interaction [46], we tested for a significant interaction between ALES and total ADAMS score. Model 4 of the SPSS PROCESS MACRO Version 5.0 [47] was used for the above procedures.

## Results

Adults with DS experienced an average of at least two ALES (*M* = 2.27, *SD* = 2.23), with about one-fifth of the sample reporting either no ALES (*n* = 26, 19.5%) or four or more ALES (*n* = 28, 21.1%). The number of ALES was significantly positively correlated with the total ADAMS score (*r* = .323, *p* < .001) but was not related to the total WHOQOL-DIS score (*r* = − .141, *p* = .104). The total ADAMS score was significantly negatively correlated with the total WHOQOL-DIS score (*r* = − .460, *p* < .001).

Among personal characteristics, intellectual disability level, age, and sex were not significantly associated with the number of ALES, ADAMS, or WHOQOL-DIS. However, results of Welch’s t-tests indicated that participants who used psychiatric medication had higher number of ALES (*M* = 3.028, *SD* = 2.613) than participants who did not use psychiatric medication (*M* = 1.990, *SD* = 2.008), *t*(51.124) = 2.159, *p* = .036; they also had higher total ADAMS scores (*M* = 14.900, *SD* = 9.886) than participants who did not use psychiatric medication (*M* = 6.066, *SD* = 6.095), *t*(45.244) = 5.019, *p* < .001. Participants utilizing psychiatric medication had lower total WHOQOL-DIS score (*M* = 63.153, *SD* = 15.649) than participants not utilizing psychiatric medication (*M* = 73.978, *SD* = 11.456), *t*(49.591) = -3.790, *p* < .001. Thus, we controlled for psychiatric medication in regression and pathway analyses.

Among environmental factors, residential setting was not significantly associated with the number of ALES, the ADAMS, or the WHOQOL-DIS. However, level of independence was significantly associated with the total ADAMS score, *F*(2, 130) = 6.432, *p* = .002, η^2^ = 0.05, and the WHOQOL-DIS score, *F*(2, 130) = 3.406, *p* = .036, η² = 0.09, and was controlled for in subsequent regression and pathway analyses. Post-hoc comparisons using Tukey-Kramer tests indicated that participants with 24-hour supervision had higher mood symptoms (*M* = 11.119, *SD* = 8.981) than participants with no supervision (*M* = 4.000, *SD* = 3.814), *M*_diff_ = 7.119, 95% CI [0.505, 13.733], *p* = .032. Participants with 24-hour supervision had lower quality of life scores (*M* = 65.063, *SD* = 15.576) than participants with minimum supervision, (*M* = 71.681, *SD* = 12.475), *M*_diff_ = 6.618, 95% CI [0.040, 13.196], *p* = .048, as well as participants with no supervision, (*M* = 80.655, *SD* = 10.039), *M*_diff_ = 15.592, 95% CI [4.990, 26.194], *p* = .002. Two dummy variables were created for use in regression and pathway analyses; participants with 24-hour supervision were the reference group. The dummy variables (no supervision and minimum supervision) were coded: yes = 1, no = 0.

**Fig. 1:**
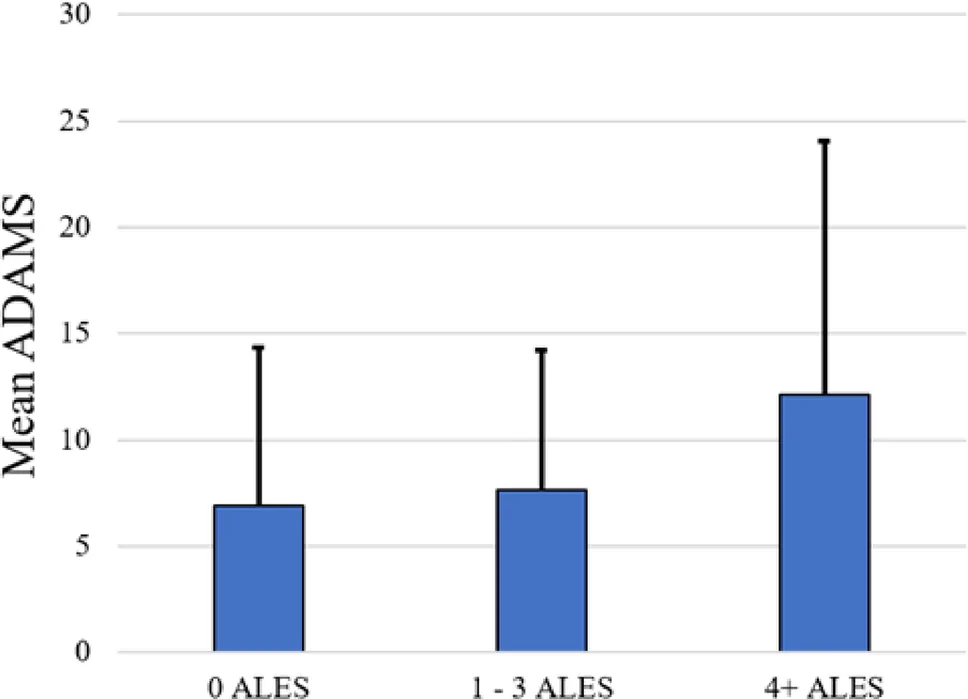
Average mood symptoms across ALES. ALES = Adverse Life Experiences. ADAMS = Anxiety, Depression, andMood Scale. *N* = 133. 0 ALES: *n* = 26. 1–3 ALES: *n* = 79. 4 + ALES: *n* = 28

**Fig. 2:**
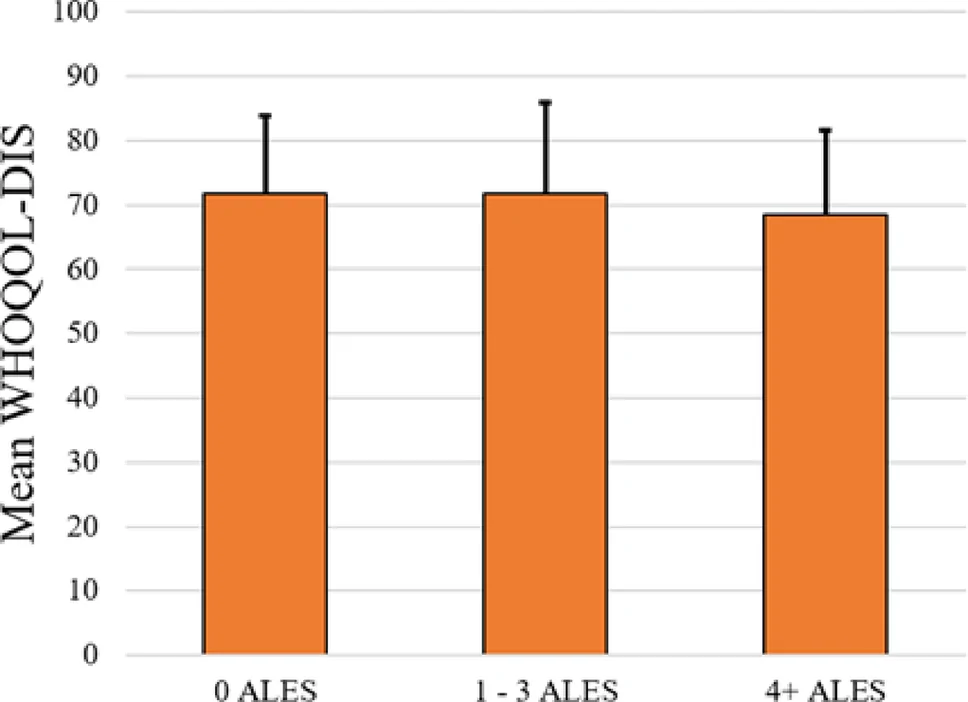
Average quality of life across ALES. ALES = Adverse Life Experiences. WHOQOL-DIS = World Health Organization Quality of Life-Disabilities Module. *N* = 133. 0 ALES: *n* = 26. 1–3 ALES: *n* = 79. 4 + ALES: *n* = 28

Figure 1 presents the average ADAMS and Fig. 2 presents the average WHOQOL-DIS scores for participants with no ALES (*n* = 26), one to three ALES (*n* = 79) and four or more ALES (*n* = 28). A Welch’s ANOVA test indicated that ALES groupings did not differ in the ADAMS score, *F*(2, 45.379) = 2.001, *p* = .147. A one-way ANOVA to test group differences in the WHOQOL-DIS score was also not statistically significant, *F*(2, 130) = 0.658, *p* = .520.

### ALES, mood symptoms and quality of life

Results from the hierarchical linear regression models are found in Table 2. Compared to an intercept-only model, the model with a constant and controls (psychiatric medication use and levels of independence) explained 15.9% of the variance in the total WHOQOL-DIS score, ∆*F*(3, 129) = 9.303, *p* < .001, ∆*R*^2^ = 0.178. Adding the number of ALES did not significantly improve model fit, ∆*F*(1, 128) = 0.812, *p* = .369, ∆*R*^2^ = 0.005. Including total ADAMS score in the final model improved model fit, with this predictor alone explaining 8.6% of the proportion of variance in the total WHOQOL-DIS score; ∆*F*(1, 127) = 14.970, *p* < .001, adjusted *R*^2^ = 0.240. Number of ALES was not a significant predictor of the total WHOQOL-DIS score (β = 0.009, *p* = .910). The total ADAMS score was significantly negatively associated with the total WHOQOL-DIS score (β = − 0.350, *p* < .001), such that a one-unit increase in mood symptoms was associated with a 0.573 unit decrease in quality of life score. In the final model, psychiatric medication use was no longer statistically significantly related to WHOQOL-DIS (β = − 0.149, *p* = .091). Participants with no supervision had a 9.873 unit increase in quality of life scores compared to participants with 24-hour supervision, holding other variables constant (β = 0.210, *p* = .020).

**Table 2:** Quality of life regressed on ALES, mood symptoms, and covariates

Model		B	SE	β	t	*p*	95% CI
1	Constant	**69.215**	**2.565**	–	**26.984**	**< 0.001**	**[64.140**, **74.290]**
	Medication	**-9.262**	**2.495**	**− 0.305**	**-3.712**	**< 0.001**	**[-14.198**, − **4.326]**
	Supervision (None)	**12.212**	**4.363**	**0.259**	**2.799**	**0.006**	**[3.580**, **20.844]**
	Supervision (Minimum)	4.681	2.698	0.160	1.735	0.085	[− 0.657, 10.020]
2	Constant	**70.118**	**2.756**	–	**25.446**	**< 0.001**	**[64.665**, **75.570]**
	Medication	**-8.793**	**2.550**	**− 0.289**	**-3.448**	**< 0.001**	**[-13.839**, − **3.748]**
	Supervision (None)	**12.278**	**4.367**	**0.261**	**2.812**	**0.006**	**[3.638**, **20.919]**
	Supervision (Minimum)	4.655	2.700	0.159	1.724	0.087	[− 0.688, 9.998]
	ALES	− 0.448	0.497	− 0.074	− 0.901	0.369	[− 1.433, 0.536]
3	Constant	**73.324**	**2.745**	–	**26.716**	**< 0.001**	**[67.893**, **78.756]**
	Medication	− 4.535	2.660	− 0.149	− 1.705	0.091	[− 9.798, 0.729]
	Supervision (None)	**9.873**	**4.193**	**0.210**	**2.355**	**0.020**	**[1.577**, **18.170]**
	Supervision (Minimum)	4.013	2.569	0.137	1.562	0.121	[− 1.071, 9.098]
	ALES	0.056	0.490	0.009	0.114	0.910	[− 0.914, 1.025]
	ADAMS	**− 0.573**	**0.148**	**− 0.350**	− **3.869**	**< 0.001**	**[**− ** 0.866**, − **0.280]**

24-hour supervision is reference category for level of independence. Statistically significant *p* < .05 in bold. *N* = 133

* ALES * adverse life experiences, *ADAMS* anxiety, depression, and mood scale

**Fig. 3:**
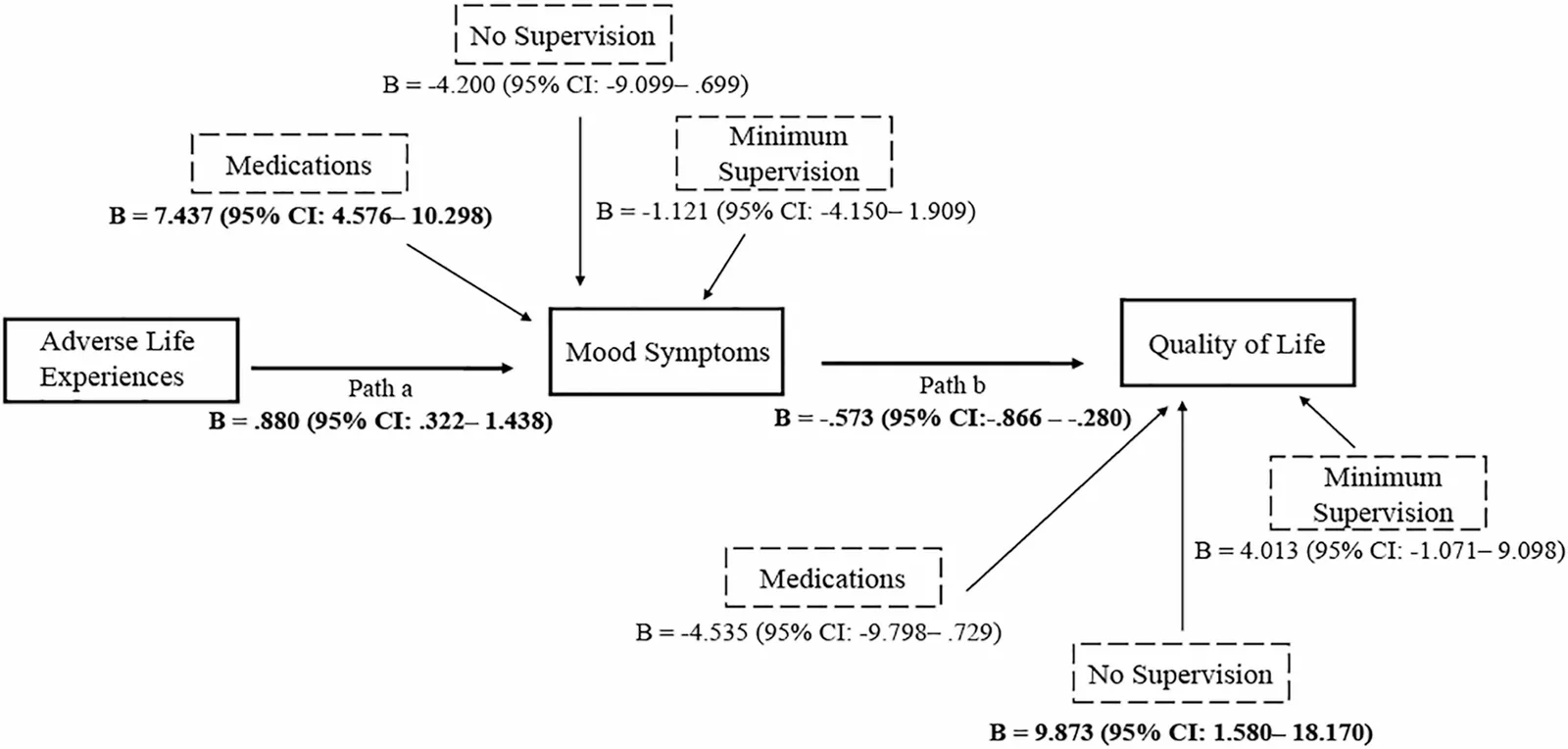
Pathway model of ALES, mood symptoms, and quality of life. 24-hour supervision is reference category for level of independence. Statistically significant path coefficients *p* < .05 in bold

Results from the pathway analysis are reported in Tables 3 and shown in Fig. 3. The first hypothesis was not supported in that the direct effect of ALES on WHOQOL-DIS was not significant (c’ path coefficient = 0.056, *p* = .910). The exposure-indirect interaction effect was not statistically significant, *F*(1,126) = 1.195, *p* = .277. The second hypothesis was supported; the path from ALES to total ADAMS score was significant, meaning participants who experienced a one-unit increase in ALES had mood symptoms that increased by 0.880 units (a path coefficient = 0.880, *p* = .002). The third hypothesis was also supported: the path from the total ADAMS score to the WHOQOL-DIS score was significant; holding ALES constant, a one-unit increase in mood symptoms reduces quality of life by 0.573 units (b path coefficient = − 0.573, *p* < .001). A percentile bootstrap confidence interval for the indirect effect based on 5000 bootstrap samples was below zero (ab path coefficient = − 0.504, 95% CI [− 1.066, − 0.094]), indicating that participants who differ by one unit on ALES are expected to differ on quality of life scores by 0.504 units lower through the effect of ALEs on mood symptoms, which in turn affects quality of life. Psychiatric medication use was associated with more mood symptoms (B = 7.437, *p* < .001). Participants without supervision had higher quality of life scores than participants with 24-hour supervision (B = 9.873, *p* = .020).

**Table 3:** Association between ALES and quality of life and indirect effect of mood symptoms

	Consequent									
		M (ADAMS)					Y (QOL)			
Antecedent		Coeff.	*SE*	95% CI	*p*		Coeff.	*SE*	95% CI	*p*
*X* (ALES)	*a*	**0.880**	**0.282**	**[0.322**,** 1.438]**	**0.002**	*c’*	0.056	0.500	[− 0.914, 1.025]	0.910
*M* (ADAMS)		–	–	–	–	*b*	− **0.573**	**0.148**	**[**− **0.866**,** − 0.280]**	**< 0.001**
*C* (Medication)	*B*	**7.437**	**1.446**	**[4.576**,** 10.298]**	**< 0.001**	*B*	− 4.535	2.660	[− 9.798, 0.729]	0.091
*C* (Supervision-None)	*B*	− 4.200	2.476	[− 9.099, 0.699]	0.092	***B***	**9.873**	**4.193**	**[1.580**,** 18.170]**	**0.020**
*C* (Supervision-Minimum)	*B*	− 1.121	1.531	[− 4.150, 1.909]	0.466	*B*	4.013	2.569	[− 1.071, 9.098]	0.121
Constant	*i*_*M*_	**5.600**	**1.562**	**[2.508**,** 8.691]**	**< 0.001**	*i*_*Y*_	**73.325**	**2.745**	**[67.893**,** 78.756]**	**< 0.001**
		R^2^ = 0.295					R^2^ = 0.269			
		F(4, 128) = 13.407, *p* < .001					F(5, 127) = 9.356, *p* < .001			

24-hour supervision is reference category for level of independence. Statistically significant *p* < .05 in bold. *N* = 133

*ALES* adverse life experiences, *ADAMS* anxiety, depression, and mood scale, *QOL* quality of life

## Discussion

This study examined the association between ALES and quality of life among adults with DS and whether mood symptoms indirectly explain at least part of this relationship. Contrary to our first hypothesis, the number of ALES symptoms was not directly associated with quality of life scores, either in bivariate analyses or after adjusting for psychiatric medication use and level of independence. As hypothesized, adults with DS who had experienced more ALES had more mood symptoms, and more mood symptoms were associated with lower quality of life. The path analysis showed a significant indirect association between ALES and quality of life through mood symptoms (− 0.504, 95% CI [− 1.066, − 0.094]). In other words, the data fit an “indirect-only” pattern [48]: ALES were linked to quality of life only through their association with mood symptoms.

Modern mediation methods do not require a significant X–Y association before testing indirect effects [41; 48–50]. Indirect-effect tests can also have greater power than total-effect tests, especially in modest samples [51–52]. Because the total association combines all pathways, including unmeasured ones that may oppose each other, a null total association can coexist with a reliable indirect association. In standardized terms, the indirect association was small, as expected for the product of two modest paths. Still, it was consistent across bootstrap resamples and remained after adjusting for psychiatric medication use and level of independence. Its practical importance is that it points to mood symptoms as a plausible point of contact between adversity history and quality of life, one that is observable and clinically addressable. Whether mood symptoms lie on a causal path from adversity to quality of life can only be established with longitudinal data.

The absence of a direct association is a central finding, and the current data cannot distinguish among several possible explanations. First, measurement may have weakened the association. The CYW ACE-Q was developed for youth and has not been validated for proxy report across the lifespan of individuals with IDD. ALES were summed into an unweighted count with no information on timing, severity, or chronicity, and recall bias over an average of nearly four decades probably added error. The WHOQOL-DIS also emphasizes autonomy, discrimination, and social inclusion [41], which current supports and opportunities likely shape as much as past experiences [4]. Earlier studies that found direct associations between ALES and quality of life relied on broad health-related measures [34–35, 53–54], so differences between instruments may partly explain our results.

Second, the null association may reflect adaptation, resilience, or protective environments. Most participants lived with family caregivers, and in adults with IDD, social support has been associated with lower odds of mental illness even when people face many stressors [55]. Long-term supportive relationships and services may protect some domains of quality of life even when mood symptoms persist. Residential setting was not associated with ALES, mood symptoms, or quality of life in this sample, but it is a crude proxy for support quality. We could not test these protective processes directly.

Finally, selection and survivor bias may have reduced the association. These participants are adults without dementia who could complete multi-day research visits with an engaged study partner. Adults whose adversity led to the most severe outcomes, such as loss of caregivers, placement disruption, regression, or earlier mortality, may be underrepresented.

The association between ALES and mood symptoms fits a substantial literature on IDD showing that life events and trauma are risk factors for psychological problems in this population [28; 56–57]. It also fits DS-specific research linking life events and ALES to psychiatric symptoms, functional decline, and DS regression disorder [25–30]. Trauma-related distress in people with IDD is often expressed through changes in mood, behavior, sleep, and social withdrawal rather than verbal report, and post-traumatic stress disorder is frequently under-recognized [57–58]. Informant-rated ADAMS scores may therefore partly reflect trauma-related presentations, not only mood symptoms in the conventional sense. Likewise, the association between mood symptoms and lower quality of life matches findings in adults with IDD that psychiatric symptoms are associated with lower quality of life in both proxy reports and self-reports [59].

Frameworks developed in the general population, such as stress sensitization [60] and allostatic load [61], offer possible ways to understand how early adversity could be linked to later mood symptoms. Given elevated inflammation and possible hypothalamic-pituitary-adrenal (HPA) axis differences in DS [62–65], these frameworks raise questions about whether adversity has distinct biological correlates in this population. However, the present study did not measure stress physiology or temporal change, so these models are best viewed as hypotheses for future longitudinal and biomarker work.

Mood symptoms accounted for almost 9% of the variance in quality of life, and psychiatric medication use and level of independence together accounted for more variance than ALES did. Medication use probably marks clinically significant psychiatric difficulties rather than acting as an independent influence. Adults receiving 24-hour supervision had more mood symptoms and lower quality of life than those with less supervision, though the direction of this association cannot be determined. Future work should examine other factors relevant to adults with IDD, including employment, social participation, adaptive behavior, and the quality of supports, ideally measured continuously rather than categorically.

These findings have important implications for clinical and organizational practices aimed at promoting optimal quality of life in adults with DS. The findings are compatible with the view, already well established in the IDD literature, that adversity histories are relevant when adults with DS present with mood or behavioral changes. Asking about ALES in that context may inform assessment, including consideration of trauma-related presentations that might otherwise be missed [58; 66–67]. Trauma-informed care frameworks proposed for IDD service organizations [68] and adapted cognitive-behavioral therapies with promising effects in adults with IDD [69] are reasonable candidates for evaluation. Gaps in coordination between mental health and IDD service systems [70] may limit access to such care.

Strengths of this study include a relatively large multisite sample of adults with DS and a quality-of-life measure designed for people with disabilities. The study also has important limitations that bear directly on interpretation of findings. The data were cross-sectional, so the path analysis cannot establish temporal ordering or causal mediation. Cross-sectional estimates of indirect effects can substantially misrepresent longitudinal processes [71]. Although ALES were referenced to lifetime, mood symptoms to the past six months, and quality of life to the past two weeks, all were reported at one visit; other orderings are plausible. Current mood or quality of life may color informants’ recall of past adversity, and mood symptoms and quality of life may influence each other (models assumed linear, unidirectional relationships).

All measures were reported by study partners, and no self-report data were collected from adults with DS. This matters most for quality of life, which the WHO defines as an individual’s own perception [3]. Agreement between proxy reports and self-reports of quality of life in adults with IDD varies across domains and is often only moderate [59, 72]. Study partners may also have incomplete knowledge of ALES, especially events that occurred before they knew the participant or that were never disclosed (e.g., abuse), leading to undercounting. Agreement on life events may also differ between family and staff informants [55]. Additionally, because one informant reported on all three constructs at the same visit, shared method variance may have inflated path associations [73].

Lifetime ALES were reported retrospectively and are vulnerable to recall bias, and the CYW ACE-Q has not been validated in adults with IDD. It yields only a count, and researchers did not collect item-level data for privacy reasons. It also omits forms of adversity experienced by adults with DS, such as diagnostic overshadowing, social exclusion, prolonged dependency, and repeated medical procedures [74–76]. ABC-DS is a research cohort focused on Alzheimer’s disease etiology. Participants were predominantly White (90%), mostly had mild or moderate intellectual disability, mostly lived with family, and were drawn from four U.S. sites. Together with the survivor bias described above, these features limit how far the findings generalize to adults with DS in general.

Future research should use longitudinal designs that measure ALES, mood symptoms, and quality of life at multiple time points, as well as cross-lagged or bidirectional models. It should also include accessible self-report measures alongside multiple informants and draw on more diverse samples. In this cohort of adults with DS, informant-reported ALES were not directly associated with quality of life, but were indirectly associated with lower quality of life through higher mood symptoms. This indirect-only pattern identifies mood symptoms as a plausible link between adversity history and quality of life in adults with DS. The findings support continued attention to adversity histories and mental health in adults with DS. They also show that longitudinal, multi-informant research that includes the perspectives of adults with DS is needed to clarify temporal ordering and identify intervention targets that could improve quality of life.

## Data Availability

The data analyzed in this study was obtained from The National Institutes of Health (NIH) National Institute on Aging (NIA) Alzheimer’s Biomarkers Consortium—Down Syndrome (ABC DS), the following licenses/restrictions apply: Applicants must complete and submit a data request form (ABC DS Data Request Form) and review and sign the ABC DS Data Use Agreement. Requests to access these datasets should be directed to ABC DS, https://www.nia.nih.gov/research/abc-ds.
